# Low Back Pain Among Nurses in a Tertiary Care Teaching Hospital at Malappuram Kerala

**DOI:** 10.7759/cureus.31622

**Published:** 2022-11-17

**Authors:** Rishad Kanakkarthodi, Binoy Edakalathur Baby, Abdul Nizar Anapattath, Jafar Kallikkattu Valappil, Afsar Afsar, Ramakrishna Pai Jakribettu, Kanniyan Binub

**Affiliations:** 1 Department of Medical Education, Muslim Educational Society (MES) Medical College, Perinthalmanna, IND; 2 Department of Pharmacology, Government Medical College, Thrissur, IND; 3 Government Health Services, Government Health Centre, Kerala, IND; 4 Department of Emergency Medicine, Muslim Educational Society (MES) Medical College, Perinthalmanna, IND; 5 General Practice, Hayath Medi Care, Kuttipuram, IND; 6 Department of Microbiology, Malabar Medical College and Research Center, Calicut, IND; 7 Department of Community Medicine, Malabar Medical College and Research Center, Calicut, IND

**Keywords:** semi-structured questionnaire, cross-sectional survey, ergonomics, nurses, low back ache

## Abstract

*Background:* Numerous nurses suffer from low back pain of various origins, which causes them to lose productivity, obtain unwanted medical reports, and sometimes even retire before their time. Age, heredity, obesity, bad posture, poor body mechanics, pregnancy, tension, personal stress, and traumatic incidents like falls or vehicle accidents are all potential causes of musculoskeletal injuries on the job.

*Methodology:* A descriptive study was done with the help of a semi-structured questionnaire to determine the burden of low back pain among nurses in a tertiary medical college. The data were entered into a Microsoft Excel sheet (Redmond, USA) before being transferred to SPSS. The frequency was expressed in proportion. A Chi-square was done to test the association.

*Result:* Among 220 nurses, 89 (40.4%) complained of mild low back pain, 86 (39.09%) complained of moderate pain, and seven (0.03%) of them had severe low back pain. Due to low back pain, among the 182 (82.7%) nurses who have low back pain, 46 of them had to take one or more days' leave from work. Thirty-six nurses have had low back pain for more than four years. As a mode of treatment, 43 nurses have taken either medicine or injection; 25 of them have taken rest; four are on Ayurvedic treatment, and 110 nurses haven’t taken any treatment.

*Conclusion:* The majority of the nurses complained of low back aches. Care must be taken to take adequate rest after prolonged standing and proper treatment. Frequent bending and using an abnormal posture must be avoided. The use of ergonomically designed chairs should be emphasized for the protection of the back.

## Introduction

Pain is defined as an uncomfortable feeling in any body part due to an illness or injury. It is said to be designed to make a person aware of the injury and protect oneself from more damage [1,2]. "Lumbago or lumbosacral pain is a kind of back pain that occurs below the 12^th^ rib and above the gluteal folds" [2,3]. The prevalence of low back pain is well-known to be a major contributor to healthcare costs and patient absences.

Among the health workers, the most vulnerable group is nurses [4]. Most of the nurses in any hospital suffer from low back pain, either due to occupational or individual reasons, and the reason for low back pain is usually multifactorial [5]. Low back pain leads to decreased labor productivity and labor force participation, which eventually leads to economic loss. It indirectly affects society and the economy of the country. According to research by Harrington and Gill, low back discomfort is the leading cause of early retirement due to illness, sick leave, job changes, and slower work speeds [4]. Nurses often forego personal hygiene and comfort in favor of lifting and transporting patients. Due to a dearth of available medical personnel and an increase in the number of patients, the majority of nurses are being forced to work overtime. Among the working population, nurses are disproportionately affected by low back pain. That is why there has been so much research done on the subject, even though the findings may be contradictory. Both the hospital administration and the nurses must be aware of low back pain since it is an occupational condition. Several nurses indicated that workplace aggression, including physical assault, contributed to their low back discomfort [6].

"Low back pain was shown to be much less common among nurses in Malaysia before they started the profession compared to later [7]. The length of time an individual has spent in the nursing profession has also been shown to be an independent risk factor for the onset of low back pain. Nurses, on average, had a prevalence of 71% for low back pain, according to the same study [6]. "Because there has been a lack of studies examining the incidence and causes of low back pain among nurses in Northern Kerala, this investigation intends to fill that gap."

## Materials and methods

In September and October of 2019, researchers from MES Medical College in Kerala, India, conducted a cross-sectional survey of the nursing staff. A convenient sample of nursing students who are involved in strenuous continuous work, including overtime, when compared to the senior and aged nursing faculties was included in the study. Nurses who had worked full-time for at least three months and who were fluent in both spoken and written English met the inclusion criteria. Nurses were not eligible if they were pregnant, had an underlying condition earlier that caused low back discomfort, including sports and road traffic accident injuries, were on extended leave, or were reluctant to participate in the trial. Special care was taken to exclude participants with mood disorders and previous history of treatment for back pain/injury. Assuming a p-value of =16.47 from prior research, the sample size was determined to be 220, or around 80% of the total population [7]. The PI collected information with the use of a semi-structured questionnaire. The frequency of low back discomfort and the factors that contribute to it were studied. The questionnaire used to capture demographic data and occupational details was self-designed, reliable, and easy to comprehend. Height, weight, body mass index, age, gender, parity, presence and intensity of low back pain, discomfort when active, and BMI were all measured. The severity of low back pain was rated using a Visual Analogue Scale (VAS; 0-10). On a scale from 0 (no discomfort) to 10 (extreme pain), 0 (no discomfort) is the baseline.

The scales of 1-3, 4-7, and 8-10 helped to grade mild, moderate, and severe pain, respectively. Various characteristics of pain, such as the mode of onset of pain, duration since the pain started, type of pain, and movement of pain, were included in the questionnaire. Radiation of pain also referred to as "pain," and migration of the pain was also included in the questionnaire. Questions on occupational details included years of experience, duration of working hours in a week, number of off-duty days in a week, number of leave applied due to low back pain, ward of posting, the shift of posting, number of patients taken care of per day, number of patients in the ward, number of nurses in the ward, and the average number of injections, dressings, and shifting of patients in a day. The questionnaire was given to the nurses according to the inclusion criteria and allowed them to fill it out on their own. The data was entered into a Microsoft Excel sheet (Redmond, USA) and analyzed using IBM Corp. Released 2017. IBM SPSS Statistics for Windows, Version 25.0. Armonk, NY: IBM Corp. The frequency was expressed in proportions, and the data was depicted using appropriate pie charts and bar diagrams. The associations of categorical variables were analyzed using the Chi-Square test. Informed written consent was taken from study samples. Ethical clearance was taken from the Institutional Ethical Clearance Review Board of MES Medical College with reference number 2019-09441.

## Results

Out of the 220 nurses, 182 of them (82.7%) were found to have low back pain, and the rest, 38 (17.3%), do not have low back pain. The study included 213 (96.81%) females and 7 (3.19%) males. The mean age of the study samples is 26.4±4.3. Among these 220 nurses, 80 (36.4%) of them are married, and 140 (63.6%) were unmarried. Among the married nurses, 41 nurses had two or fewer children, six nurses had more than two children, and 33 nurses had no children. Sixty-seven nurses had a duration of working less than 48 hours, and the rest 153 nurses had more than 48 hours per week. One hundred twelve nurses were posted in General wards, 61 of them in the intensive care unit (ICU), 30 in Operation Theatres, 16 in the Emergency Department, and one in the Cath Lab.

The mode of onset of low back pain in 116 nurses was gradual in onset, and 66 of them had a sudden onset. Among 220 nurses, 89 complained of mild low back pain (scale of 1 to 3), 86 complained of moderate pain (scale of 4 to 7), and seven had severe low back pain (scale of 8 to 10). Sixty-nine nurses had a shooting type of pain, 44 of them had a dull aching type of pain, 35 had a throbbing type of pain, and 33 had a stabbing type of pain. In studying the movement of pain, 85 nurses had radiation of pain to the buttocks and back of the thigh, 29 nurses had migration of pain, 23 nurses have referred pain, and 44 of them do not have movement of the pain. Due to low back pain among the 182 nurses who have low back pain, 46 of them had to take leave for one or more days from the work, and 136 of them did not take any leave. Thirty-six nurses have had low back pain for more than four years and 146 of them have had low back pain for less than four years. As a mode of treatment, 29 nurses had undertaken physiotherapy, 43 nurses had taken either medicine or injection, 25 of them take rest, four are on Ayurvedic treatment, and 110 nurses haven’t taken any treatment. Table 1 depicts the demographic data of the participants in the study.

**Table 1:** Frequency and percentage of different variables LBP: Low back pain

Variable		Frequency	Percentage
Presence of back pain			
Yes		182	82.7
No		38	17.3
Gender			
Females		213	96.81
Males		7	3.19
Age			
Less than 25 years		103	46.8
More than 25 years		117	53.2
Marital status			
Married		80	36.4
Unmarried		140	63.6
Parity			
1 Child		32	14.55
2 Children		9	4.09
3 Children		5	2.27
4 Children		1	0.45
No children		173	78.64
Duration of working hours per week			
Less than 48 hours		67	30.5
More than 48 hours		153	69.5
Ward of posting			
Ward with emergency procedures		16	7.3
Regular wards		112	50.9
Observation wards		92	41.8
Mode of onset of LBP			
Gradual		116	52.7
No		38	17.3
Sudden		66	30
Severity			
Mild		89	48.9
Moderate		86	47.3
Severe		7	3.8
Type of pain			
Shooting		69	37.91
Dull aching		45	24.73
Throbbing		35	19.23
Stabbing		33	18.13
Movement of pain			
Radiation		86	47.25
Migration		29	15.93
Referred		23	12.64
No movement		44	24.18
No. of leave applied due to LBP			
1 more leave taken		46	25.27
No leave taken		136	74.73
Duration since pain started			
Less than 4 years		146	80.22
More than 4 years		36	19.78
Treatment			
Physiotherapy		29	15.93
Medicine, Injection		43	23.63
Rest		25	13.74
Ayurvedic treatment		4	2.2
Not taken		81	44.5

The results of a study examining the correlation between a variety of characteristics and lumbar spine pain, as determined by the Chi-Square test, are shown in Tables 2, 3.

**Table 2:** Association of variables with low back pain LBP: Low back pain BMI: Body mass index

		Presence of LBP			Chi-Square Value	P
Variable		No	Yes	Total		Value
Gender						
	Female	35	178	213		
	Male	3	4	7	3.31194	0.05
	Total	38	182	220		
Marital status						
	Married	19	61	80		
	Unmarried	19	121	140	3.69113	0.055
	Total	38	182	220		
BMI						
	Underweight	12	43	55		
	Normal	14	73	87		
	Overweight	5	26	31	2.24876	0.08
	Obese	6	31	37		
	Very obese	1	9	10		
	Total	38	182	220		
Ward of posting						
	Ward with emergency procedures	2	14	16		
							Regular wards	28	84	112	9.67171	0.008
	Observation wards	8	84	92		
	Total	38	182	220		
	Transportation to work						
	Bus	19	89	108		
	Self-vehicle	5	11	16		
	Taxi	0	4	4	3.30242	0.27
	Walking	14	78	92		
	Total	38	182	220		
Duration of exercise per week						
								1 hour	2	6	8		
15 mins	1	0	1				
2 hours	0	4	4				
3.5 Hours	0	1	1	3.642	0.16		
30 mins	3	6	9				
No exercise	32	165	197				
Total	38	182	220				
Wearing high heels or not						
	No	38	179	217		
	Yes	0	3	3	0.63503	0.426
	Total	38	182	220		
Parity						
	1 Child	6	26	32		
	2 Children	2	7	9	1.24689	0.97
	3 Children	1	4	5		
	4 Children	0	1	1		
	No children	29	144	173		
	Total	38	182	220		
Working hours per week						
								Less than 48 hours	8	59	67		
More than 48 hours	30	123	153	1.91711	0.166		
Total	38	182	220				

**Table 3:** Association of duration since pain with severity of pain

Duration since pain started	Severity of pain			Total	Chi-square value	P-Value
	Mild	Moderate	Severe			
Less than 4 years	66	78	2	146	19.7796	0.000
More than 4 years	23	8	5	36		
Total	89	86	7	182		

Based on the data shown in Table 2, we may conclude that males and females have similar rates of low back discomfort. Low back pain among nurses does not discriminate based on marital status, as seen in Table 2. Body mass index (BMI) is unrelated to lumbar discomfort is made very obvious. When dealing with low back discomfort, how you go to work does not matter. The length of an exercise session does not correlate with the occurrence of low back discomfort. Low back discomfort is the same whether you wear high heels or not. No correlation between working hours and low back pain was found, and the chart also shows that parity does not affect low back pain. A statistically significant (p=0.008) correlation between the ward of posting and the occurrence of low back discomfort is shown in Table 2. Table 3 shows that there is a statistically significant (p=0.000) correlation between the length of time after pain onset and low back pain intensity.

## Discussion

Participants include 220 nurses from a tertiary care teaching hospital in northern Kerala. In the population studied, 82.7% reported having low back discomfort at some point. The prevalence of low back pain in other research on the subject is close to 84.5 percent [7]. There were mostly women (96.81%) in the sample of nurses. The mean age of the study samples is 26.44.3, and most of them belong to the age group of 20-30. One hundred and seventeen nurses had a duration of nursing experience of fewer than 10 years (80.45%). Most nurses were normal in BMI (39.5%). 36.4% of the nurses were married, and 58.75% of them had one or more than one child.

A major group of nurses (69.54%) worked for more than 48 hours a week. About half of the nurses are posted in regular wards, and others in wards with emergency procedures and observation wards. It is also noted that most nurses had a gradual onset of low back pain. About an equal number of nurses complain of having mild (48.9) and moderate (47.3) back pain. While enquiring about the type of pain, most of the nurses had a shooting type of pain (37.91%), followed by dull aching (24.73%), throbbing (19.23%), and stabbing (18.13%) types of pain. Furthermore, many nurses report pain radiating to the back of the thigh and leg and, in some cases, the buttocks. Some say they had migratory pain (15.93%) and referred pain (12.64%). Only 24.18% reported no pain or movement. About one-fourth of the nurses (25.27%) had to take one or more days of leave due to low back pain, whereas the rest of them had not taken any leave due to low back pain.

This research shows that 178 out of 213 females (83.56%) had low back discomfort, whereas four out of seven men (57.14%) did so. This research found no statistically significant link between gender and LBP. The prevalence of low back pain in men has been reported to be lower than among females several studies. A Nigerian hospital allegedly investigated the difference in low back pain rates between men and women [8]. While mechanical disadvantages like sprains and strains are more common in women than men, some researchers believe that gender variations in anatomy, physiology, and structure are to blame for the disproportionate number of female casualties [9-11]. Low back discomfort was experienced by 61 (76.25%) of the 80 married nurses and by 121 (86.42%) of the 140 single nurses. Thus, there is no statistically significant correlation between marital status and the occurrence of low back pain (p=0.055). Finding no statistically significant link between BMI and lumbar discomfort, this study concludes that obesity and back pain are unrelated. Chan Siok Gim discovered no connection between body mass index and the incidence of low back discomfort (p=0.221) [7]. In contrast, body mass index (BMI) has been linked to experiencing low back discomfort in previous research.

Nurse posting wards are classified into three types: Wards with Emergency Procedures, Regular Wards, and Observation Wards with emergency procedures include the emergency department and labor room. Regular wards are the general wards in the hospital, like the surgery ward, medicine ward, ENT ward, etc. Observation wards include ICUs, operation theatres, and labor rooms. From the data, it is clear that nurses working in the observation wards are more prone to low back pain (91.30%), followed by wards with emergency procedures (87.50%) and regular wards (75.00%). Nurses in ICU and OT are required to stand for long periods of time, are more prone to frequent bends, and transport and carry patients sometimes, as most of them will not be in a healthy condition for their daily routines [12]. Nurses who are working in ICUs are more susceptible to low back pain compared with nurses in other critical care units. Similar results were obtained in 22 South Korean hospitals where 90.3% of patients had back pain [13]. Nurses were asked about their mode of transportation to work, and almost equal prevalence was seen in those who came walking (84.78%) and those who came to work by bus (82.40%). As a result, transportation to work with low back pain has little significance (p-value = 0.270). Regular exercisers among nurses had a lower prevalence of low back pain (73.1% vs. 83.75%) than those who did not exercise at all (p=0.160). Thus, the current research finds no statistically significant link between exercise duration and low back discomfort. Chan Siok Gim found no evidence that participating in sports regularly is beneficial to health (p-value = 0.058) [7]. There was no statistically significant link found between activity and back pain, although those who did not exercise at all were more likely to have low back discomfort [14]. Low back discomfort affected nurses without children and those with children were found to be 83.23% and 80.85%, respectively (p=0.97). 

While this research did find a correlation between having children and having more instances of low back pain, it also found that the risk rose in proportion to the number of children a nurse had. One research study indicated that 35.5% of nurses who have had between one and three children have low back discomfort on a regular basis, and that number rises to 63.5% among those who have had more than four children in their careers [7]. This is consistent with the report that 51.5% of nurses who have delivered many babies have LBP [15]. Back discomfort was cited as a common complaint among female nurses, and it was often associated with pregnancy and labour [16]. When comparing nurses who worked fewer than 48 hours per week to those who worked more than 48 hours per week, we found that 88.05% of the former group had low back discomfort, whereas only 80.39% of the latter group did (p = 0.16). This study's findings disprove the hypothesis that nurses are more likely to get low back pain if they work longer hours. Studies have also indicated that nurses who work between 31 and 40 hours per week are more likely to have low back pain (21.8%) than their counterparts who work between 10 and 20 hours per week (1.8%). Of all nurses, those who put in more than 50 hours a week were the most likely to complain of low back pain (50%). Researchers also observed that nurses who worked more than 20 hours per week had low back pain [17].

This research also evaluated the influence of time since pain onset on the severity of low back pain (Table 3). Participants who reported fewer than four years of low back pain reported 45.20 percent light discomfort, 53.42 percent moderate pain, and 1.36 percent severe pain. According to the total number of people who reported having low back pain for more than four years, mild, moderate, and severe low back pain, respectively, were detected in 63.8%, 22.2%, and 13.8% of these people (p-value = 0.000). The intensity of nurses' low back pain grows gradually with the length of time since the discomfort first appeared, as shown by the statistics. Data analysis shows that low back pain is more severe among nurses who have been complaining of it for more than four years compared to those who have been complaining for less than four years.

The study's limitations are that it has a limited sample size, and a very limited population-based trial was done. The study involved a very small questionnaire where more questions can be included. The study has limits with the etiological factors of lower back pain, which is multifactorial, and hence, various etiologies can be studied in future research. 

## Conclusions

A tertiary care teaching hospital's nursing staff participated in this research to learn more about the prevalence of low back pain in the workplace. Numerous potential causes of nurses' low back discomfort have been considered in the research. The degree of low back pain was shown to be significantly related to the length of time since the discomfort first appeared (p = 0.0001). However, it should be noted that women have a greater risk of low back pain than men do because of mechanical disadvantage and parity, even if the research did not provide data to indicate a relationship between gender and low back pain.

Multiple contributors to nurses' low back discomfort were found in this investigation. Ward of posting was shown to be connected with the existence of low back pain, although gender, marital status, body mass index, mode of transportation to work, exercise time per week, number of children, and number of hours worked per week were not. It was also discovered that the length of time a person had been experiencing pain was linked to how severe their low back pain was. Evidence-based treatment should be taken for the exact etiology. Adequate rest must be ensured who have chronic low back aches. The use of ergonomically designed chairs is the need of the hour. Systematic training should be given to take care of the postures.
